# Variation in Raw Milk Microbiota Throughout 12 Months and the Impact of Weather Conditions

**DOI:** 10.1038/s41598-018-20862-8

**Published:** 2018-02-05

**Authors:** Nan Li, Yuezhu Wang, Chunping You, Jing Ren, Wanyi Chen, Huajun Zheng, Zhenmin Liu

**Affiliations:** 1 State Key Laboratory of Dairy Biotechnology, Shanghai Engineering Research Center of Dairy Biotechnology, Synergetic Innovation Center for Food Safety and Nutrition, Dairy Research Institute, Bright Dairy & Food Co., Ltd., Shanghai, 200436 China; 2 Shanghai-MOST Key Laboratory of Health and Disease Genomics, Chinese National Human Genome Center at Shanghai, Shanghai, 201203 China; 3 Key Laboratory of Reproduction Regulation of NPFPC, Shanghai Institute of Planned Parenthood Research, IRD, Fudan University, Shanghai, 200032 China

## Abstract

Milk microbiota has a great influence on the safety and quality of dairy products. However, few studies have investigated the variations of bacterial composition in raw milk. In this study, raw milk samples were collected in 12 successive months, and their bacterial compositions were determined by 16 S rRNA gene sequencing. The highest diversity of bacterial composition was detected in June, while the lowest was in December. *Firmicutes*, *Proteobacteria* and *Actinobacteria* were the most abundant phyla and exhibited a counter-balanced relationship. *Pseudomonas*, *Lactococcus* and *Acinetobacter* were the most prevalent genera (>1%), and a tiny core microbiota (*Acinetobacter* and *Pseudomonas*) was observed. Temperature and humidity were the determining factors for most variation in bacterial compositions at both the phylum and genus levels. Higher abundances of *Pseudomonas*, *Propionibacterium* and *Flavobacterium* were correlated with low temperature. Furthermore, *Pseudomonas*/*Propionibacterium* and *Lactobacillus*/*Bifidobacterium* were two pairs of genera that had synergistic effects. Associations between the microbiota and milk quality parameters were analyzed. The abundances of *Propionibacterium and Pseudoalteromonas* were negatively correlated to total bacterial count, which meant that they helped to maintain milk quality, while a series of environmental microorganisms contributed to the spoilage of raw milk.

## Introduction

Milk and manufactured dairy products are important components of a healthy diet and are among the most frequently consumed foods. A highly nutritious food, milk is composed of proteins, fats, carbohydrates, vitamins and minerals, providing an ideal environment for the growth of microorganisms^1^. Currently, the demand for safe and high-quality food has become the focus of public attention. While milk used to be considered one of the healthiest foods with the highest quality, current food recalls and foodborne illness outbreaks have indicated the need for an in-depth investigation^2,3^.

Bacterial contamination of dairy products is a major concern throughout the world^4^, as dairy production and consumption have expanded extensively during the last decade. Raw milk is known to usually harbor complex microbial communities containing diverse microorganisms^5,6^. The indigenous microflora in raw milk has a direct impact on the subsequent development of dairy products^7^. Microorganisms could not only affect milk quality and shelf life but also produce extracellular lipases and proteases, which could result in spoilage^8–10^. In addition, abnormal microbial compositions in milk could cause health problems, in that the consumption of raw milk contaminated with pathogens can lead to severe illness^11,12^.

High-throughput sequencing has been revolutionarily developed for the analysis of microbial ecology and has provided detailed insights into a series of raw milks and dairy products, such as goat milk^13^, buffalo milk^14^, naturally fermented milk^15^, artisanal cheeses^16^ and breast milk^17^. In addition, factors related to raw milk processing, including storage tanks^18^, transportation vehicles^19^ and pasture altitude^20^, have been assessed. However, the role of weather conditions on the microbial communities in raw milk has not yet been studied.

On-farm knowledge of the bacterial communities in raw milk is important for the identification of bacterial composition and assessment of its regularity. Descriptions of microbial composition and distribution would provide necessary information for the risk assessment and quality control of raw milk throughout the year. In addition, it ensures the high performance and traceability of the subsequent pasteurization and processing. To get a better understanding of indigenous bacterial compositions and to guarantee the safety and quality of raw milk and dairy products, we investigated the bacterial communities of raw milk from ten dairy farms in Shanghai (China) with high-throughput sequencing technology. Specifically, we investigated the variation in the bacterial communities throughout one whole year. The effects of temperature and humidity were considered in a correlation analysis with the bacterial communities.

## Results

### Bacterial diversity in cow milk

From July 2015 to June 2016, we collected cow milk samples each month from 10 farms; a total of 112 samples were collected. A total of 2,503,353 (7,780~34,904) high-quality 16 S rRNA gene reads were obtained by high-throughput DNA sequencing that met quality-filtering requirements. To compare the samples without statistical bias, the minimal read number 7,780, as a normalization size for each sample, was chosen for calculation of the diversity indices. The operational taxonomic unit (OTU), or group of 16 S rRNA genes, was set at 97% similarity to estimate the diversity of the bacterial community. After all samples were grouped from the 12 months, a total of 12,898 OTUs (average 2,755, from 863 to 4,469) were obtained. Good’s coverage was an average of 98.2% (97.4%~98.7%, Table 1), which meant that the sequencing depth was sufficient for the microbiota investigation of cow milk over 12 months.

**Table 1:** Bacterial richness and diversity estimates at genetic distances of 3%.

Month	OTUs	Coverage	SEM	ACE	SEM	Chao	SEM	Shannon	SEM
Jan	2570	0.982	0.003728	5424.179	151.447	4212.251	86.84022	3.261	0.285948
Feb	2183	0.984	0.005885	5123.543	200.1453	3818	151.0531	3.3	0.418796
Mar	3975	0.976	0.006953	9033.945*	286.1278	6998.382*	180.4165	4.843	0.301788
Apr	2611	0.983	0.004218	6585.588	178.1783	4788.6	95.91588	3.583	0.180692
May	1999	0.985	0.003139	6856.097	208.7313	4330.616	94.78375	3.015	0.089325
June	4469	0.977	0.004804	7941.831**	205.4795	6749.65**	130.5882	5.426	0.233656
July	3516	0.974	0.006174	8988.738**	256.194	6607.327**	114.5416	4.944	0.320875
Aug	2751	0.985	0.005982	5272.712	194.8767	4339.898	142.9341	4.245	0.256475
Sept	2720	0.984	0.00429	5251.533	143.3089	4236.942	107.8009	4.002	0.205467
Oct	2260	0.985	0.004677	5694.692	215.2667	4147.889	128.0317	3.206	0.140235
Nov	3139	0.984	0.003624	4722.395	220.7119	4703.58	107.3771	4.203	0.472844
Dec	863	0.987	0.004162	2841.298	210.8459	1818.37	110.98	2.961	0.432634

“*”indicated p < 0.05, “**”indicated p < 0.001.

**Figure 1:**
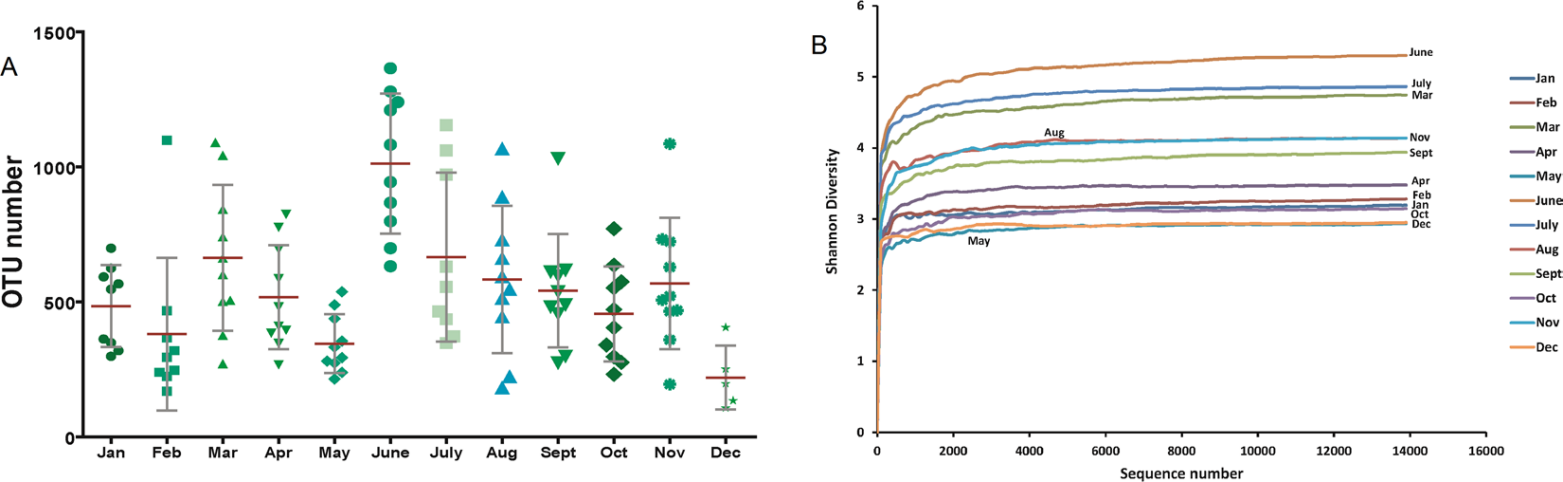
(**A**) Scatter plots showing OTU numbers with respect to total sequences obtained from 16 S rRNA genes associated with months. The error bars were the mean with SD. (**B**) Shannon diversity curve of bacterial community derived from 12 months.

Phylogenetic and taxonomic assessments of the 16 S rRNA V3–4 regions revealed that the diversity of bacterial populations in the raw milk greatly varied among different months. Based on the OTU evaluation, the total estimated bacterial richness per sample differed between months. The milk collected in March, June and July contained higher species richness according to the ACE and Chao index (Table 1). Similarly, a higher number of OTUs was observed in milk examined in these three months (Fig. 1A). There was an average of 29.3% of OTUs (1,158 OTUs) uniquely present in March, June and July, and other months had an average of 19.0% total unique OTUs. The highest number of OTUs was detected in June, while the lowest was in December.

The Shannon index showed that the samples from December had the lowest diversity, while the highest diversity was from the June samples (Shannon: December/June = 2.96/5.43, p < 0.01, Fig. 1B).

### Variable richness and high diversity of bacterial populations in cow milk depending on month

**Figure 2:**
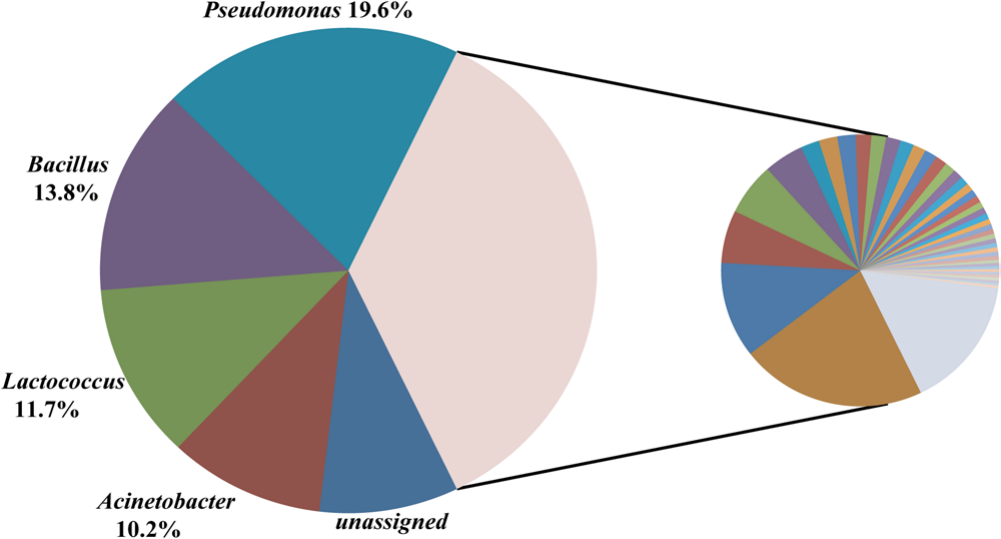
Illustration for four major abundant genera. All genera were showed in the pie chart. The left pie showed four major abundant genera and unsigned sequences which were not classified to genus; the right pie showed other 781 genera.

An RDP (Ribosomal Database Project) analysis of 112 samples assigned 98.3% (SD = 0.014) of the 16 S rRNA genes to 33 phyla and 91.0% (SD = 0.039) of the 16 S rRNA genes to 785 genera, respectively. A total of 70% or more of the taxa detected represented less than 1% of the abundance in all OTUs. At the phylum level, 4 of 33 phyla were abundant in cow milk, *i.e*., 40.8% (SD = 0.217) of 16 S rRNA genes were assigned to *Firmicutes*, 39.0% (SD = 0.141) to *Proteobacteria*, 9.40% (SD = 0.058) to *Actinobacteria* and 7.47% (SD = 0.032) to *Bacteroidetes*. Among the 785 genera, four were abundant, *i.e*., 19.6% (SD = 0.122) of 16 S rRNA genes were *Pseudomonas*, 13.8% (SD = 0.096) were *Bacillus*, 11.7% (SD = 0.078) were *Lactococcus* and 10.2% (SD = 0.088) were *Acinetobacter* (Fig. 2). The remaining 35.6% of the 16 S rRNA genes were assigned to 781 other bacterial genera, demonstrating the high diversity of the cow milk bacterial community.

**Figure 3:**
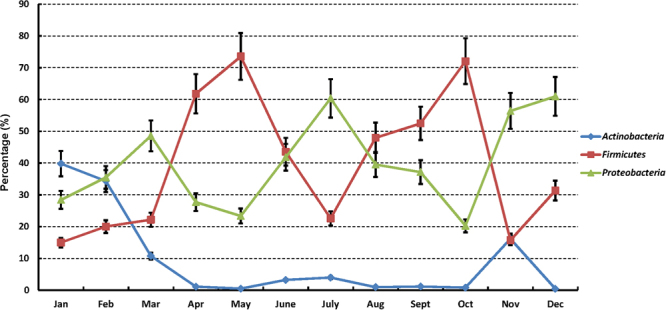
Microbiota variation of three phyla in 12 months. These three phyla showed the difference by the months which might be caused by the temperature and humidity changes. The three phyla were *Actinobacteria* (0.4%~39.8%), *Firmicutes* (15.0%~73.6%) and *Proteobacteria* (20.3%~61.0%).

Based on phylum information (Fig. 3), which was derived from the classification of OTU representative sequences by the RDP classifier, *Firmicutes*, *Proteobacteria* and *Actinobacteria* showed large differences (p < 1E-20) by month. In January, February, March and November, the abundance of *Actinobacteria* was over 10% of all phyla and was obviously greater (p < 1E-50) than that in other months. In April, May, June, August, September and October, the abundance of *Firmicutes* was up to 40%~70% of all phyla. In addition, the abundance of *Proteobacteria* was almost 60% of all phyla in July, November and December. The total proportion of these three phyla was up to 80%~95%.

**Figure 4:**
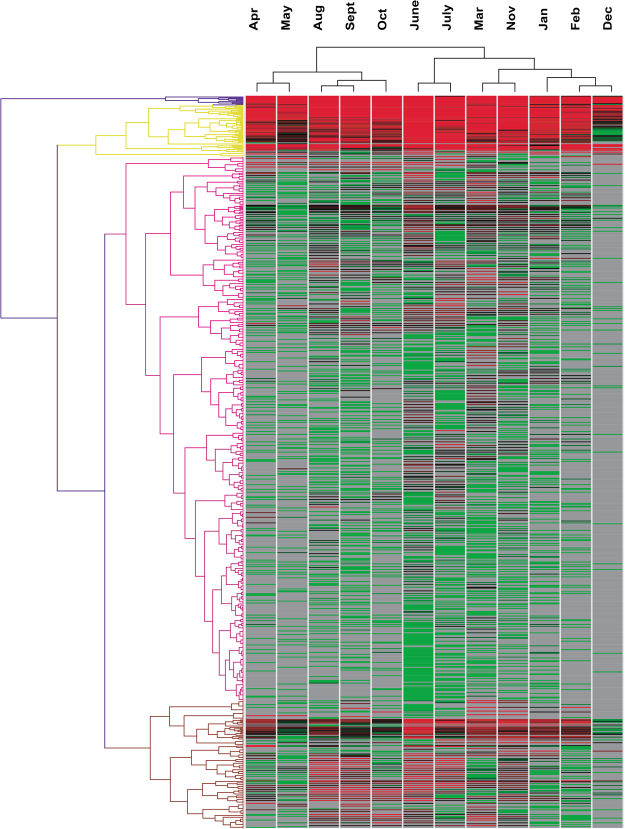
Heatmap illustrating different genera percentage by 12 months. Hierarchical cluster was calculated for the month and genera. More details of different genera were listed on the Supplementary Tables S2–S12.

**Figure 5:**
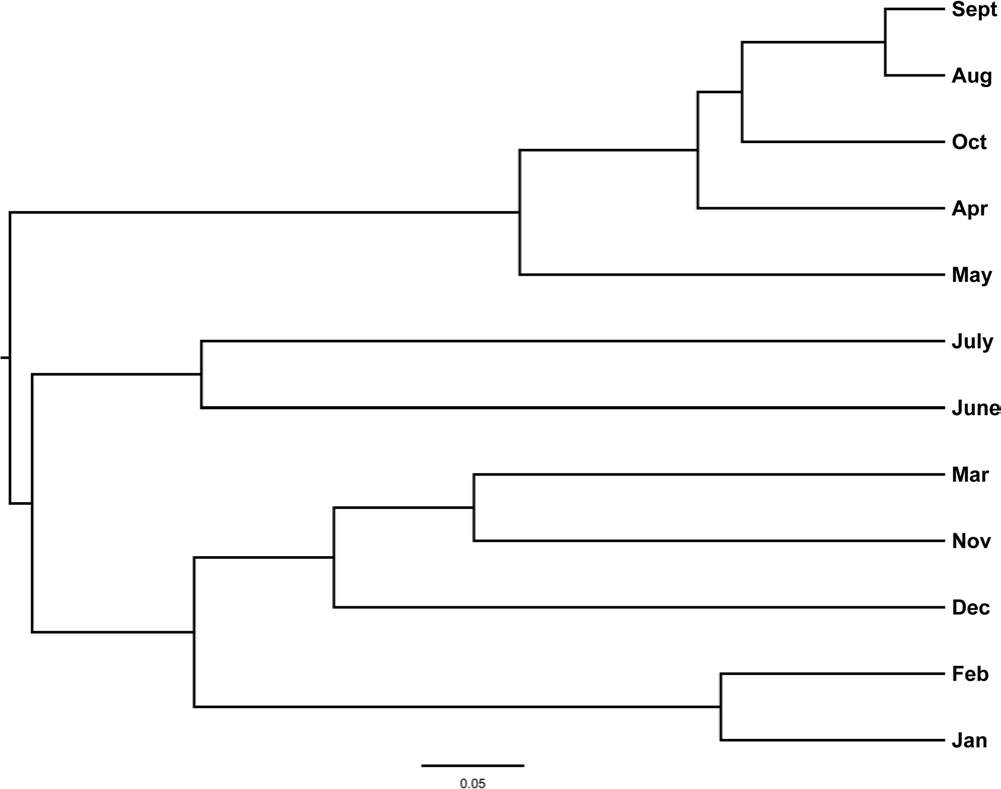
The tree showing the cluster of bacterial community using the Yue & Clayton theta similarity coefficient based on the sequences matrix.

Information regarding genus prevalence for the samples from each month was illustrated with a heatmap by hierarchical cluster analysis (Fig. 4), which showed a similar topological structure of monthly distribution with the results based on a sequence matrix (Fig. 5**)**. Adjacent months were usually located in the same clade, such as July and June, September and August, January and February, which meant that the microbiota compositions were similar between adjacent months. It was also shown that the samples could be grouped into two clades according to the season. Spring and autumn formed one clade, while summer and winter belonged to the other clade. This effect might be partly attributed to temperature and humidity changes (Table S1).

**Figure 6:**
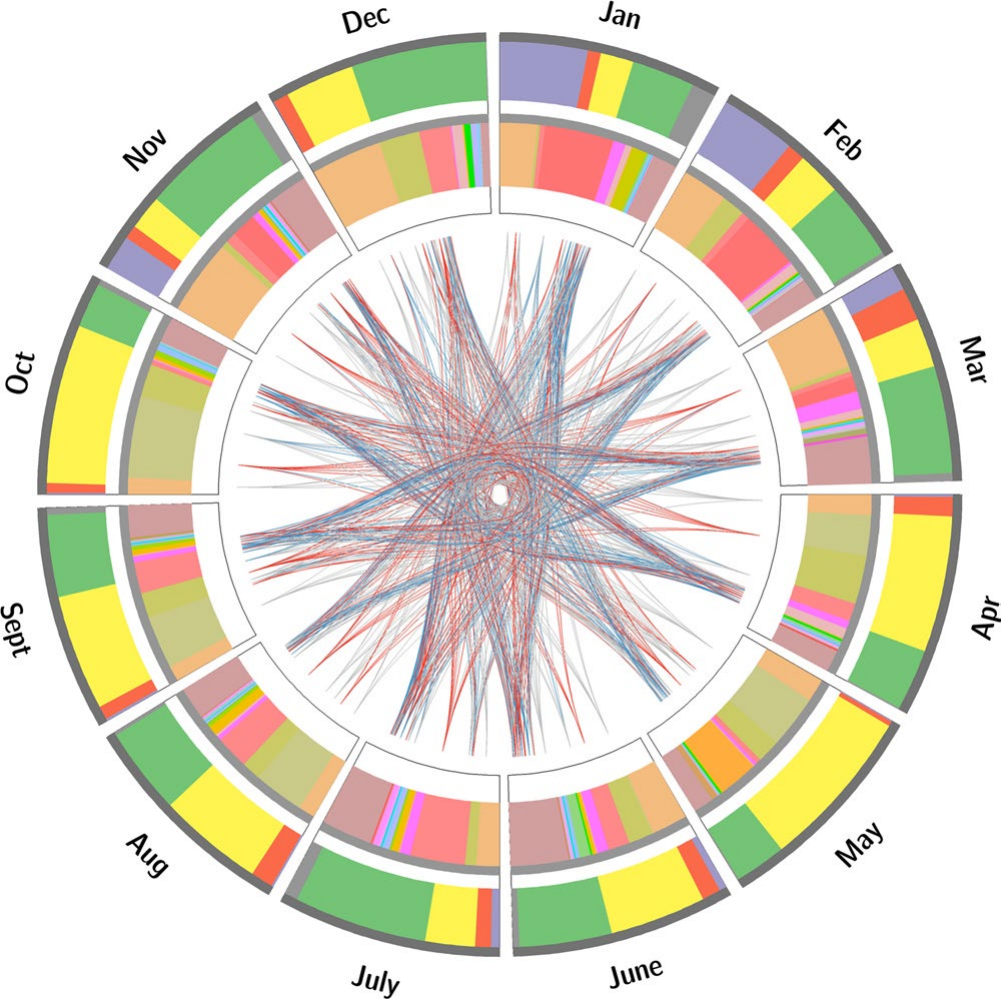
The complex illustrations showing the major phyla and genera in 12 months and the significant differences between major genera among 12 months. (1) The outer circle showed the phylum level and percentage of major phyla, purple: *Actinobacteria*, red: *Bacteroidetes*, yellow: *Firmicutes*, green: *Proteobacteria*, grey: other phyla. (2) The inner circle showed the genus level and percentage of major genera which accounting for at least 1% and had significant difference (Q-value < 1E-5) between months. (3) The lines linked the different months showed the major genera difference, red line: fold change >50, blue line: 50≥ fold change >10, grey line: 10≥ fold change >2, and more details were showed in the supplementary Tables S2–S12.

An analysis of the 16 S rRNA gene sequences showed that three genera were most prevalent (>1%) in all 12 months, which were *Pseudomonas*, *Lactococcus* and *Acinetobacter*. *Pseudomonas* represented an average of 12.0% (SD = 0.039) in April-October (average temperature of 22.8 °C) and 32.4% (SD = 0.090) in November-March (average temperature of 8.8 °C). *Acinetobacter* represented an average of 19.4% (SD = 0.079) in June-September (average temperature of 25.7 °C) and 6.0% (SD = 0.031) in October-May (average temperature of 12.6 °C). *Lactococcus* was abundant throughout the year and varied irregularly between adjacent months. Many other significant differences were present in the bacterial communities between months (Fig. 6, Tables S2–S12, p < 1E-5). Considering only the genera whose proportion was greater than 0.1% in one of two compared months, we could reveal a total of 122 genera showing significant differences between months. That meant most genera (84.5%) showed no difference between months, and these genera were a low proportion of the total (summing up to average of 10.7%, 2.7%~18.5%). Therefore, the differences were caused by the high abundance genera. When the criterion for genus proportion was raised to 1%, only 27 genera had significant differences, and those genera occupied an average of 81.8% (68.1%~95.8%) of the total bacterial population (Table S13). Thus, the subsequent analysis was focused on these 27 major genera.

Despite these differences of bacterial communities in cow milk samples, a tiny core milk microbiome was still present at the genus level. Only two genera (*Acinetobacter* and *Pseudomonas*) belonging to the *Proteobacteria* phylum were detected in all 112 cow milk samples examined. Notably, *Lactococcus* was not a part of the core microbiome, although it was present in relatively high proportions in some of the cow milk tested; *Lactococcus* was entirely absent from one sample and was therefore not included in the core. When focusing on the OTU (species) level, no core species was found in all samples, reflecting the high diversity of microorganisms in cow milk.

### Correlations between bacterial composition in cow milk and weather conditions

**Figure 7:**
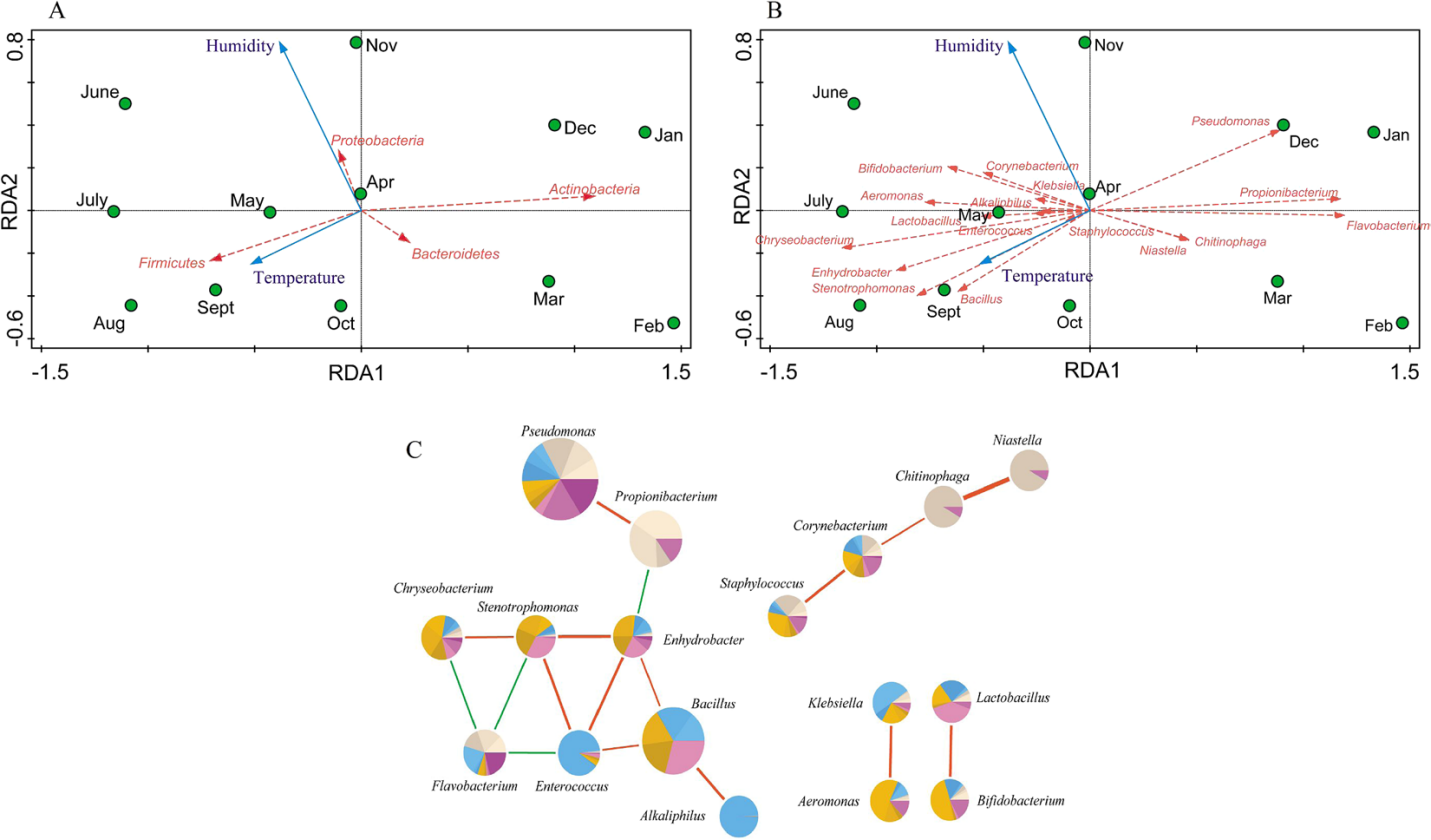
(**A**) Redundancy analysis (RDA) of the relationship between the environment factors and the relative abundance of bacterial phylum of 12 months’ samples. (**B**) Redundancy analysis (RDA) of the relationship between the environment factors and the relative abundance of bacterial genus of 12 months’ samples. (**C**) Correlation of 17 major genera. Line: orange-red (positive relation) and green (negative relation). Line weight: correlation strength. Circle size represented the reads numbers. The pie chart presented relative proportion in 12 months, seashell series: 1–3 months, blue series: 4–6 months, orange series: 7–9 months, orchid series: 10–12 months.

Redundancy analysis (RDA) indicated that temperature and humidity (Table S1) were the determining factors for most of the variation in the bacterial compositions of the samples over 12 months (Fig. 7A,B). Higher abundance of phylum *Actinobacteria* was correlated with low temperature, while higher abundance of *Firmicutes* was correlated with high temperature. Higher abundance of phylum *Bacteroidetes* was correlated with low humidity, while higher abundance of *Proteobacteria* was correlated with high humidity. At the genus level, higher abundances of *Pseudomonas*, *Propionibacterium* and *Flavobacterium* were correlated with low temperature, while the abundances of *Niastella* and *Chitinophaga* were correlated with low humidity. In addition, higher abundances of most genera were correlated with high temperature and high humidity. Meanwhile, correlation patterns were also analyzed among the 785 genera. A Spearman algorithm analysis validated that 17 of the 27 major genera had strong correlations (Fig. 7C, Table S14, p < 0.01), with parameters set as coefficient >0.68 or <−0.68, which were considered to represent strong or high correlations^21^. Genus *Flavobacterium* was negatively correlated with *Enterococcus*, *Chryseobacterium* and *Stenotrophomonas*, while genus *Propionibacterium* was negatively correlated with *Enhydrobacter*. Other genera exhibited positive correlations, such as *Pseudomonas* and *Propionibacterium*, *Lactobacillus* and *Bifidobacterium*.

### Correlations between microflora composition and raw milk quality parameters

According to the correlation analysis among 785 genera, many genera exhibited significant correlations with raw milk quality parameters (Table S15), such as somatic cell count (SCC), total bacterial count (TBC), heat-resisting spores, psychrotrophic bacteria, milk protein content and milk fat content.

There were five genera (*Azospira*, *Brachymonas*, *Chryseobacterium*, *Cloacibacterium* and *Sulfurospirillum*) showing extremely significant correlations with SCC (p < 0.001), indicating that these bacteria had a great influence on the quality of raw milk and the health status of milking cows.

*Stackebrandtia*, *Teredinibacter* and *Gemella* were the top three genera positively correlated to TBC (p < 0.001), coinciding with the factor that they were also negatively correlated with milk fat content (p < 0.05).

Dozens of genera were demonstrated to be significantly correlated with heat-resisting spores (p < 0.001), while *Methylonatrum* showed extreme significance.

Only *Brochothrix* was found to be positively correlated with psychrotrophic bacteria (p < 0.01). To our surprise, the correlation between *Pseudomonas* and psychrotrophic bacteria was not significant (p = 0.22).

Quite a few bacteria were negatively correlated with protein content, such as *Bacteroides*, *Streptococcus*, *Agrococcus*, and *Ornithinimicrobium* (p < 0.05), which meant that milk protein decreased with the increased abundance of these bacteria.

In addition, *Atopobium* was found to be the most significantly contributor to the degradation of milk fat (p < 0.01).

### Microbial function prediction

The microbial functions of each month were predicted by PICRUSt software. Using strict parameters, we found no functional difference among some consecutive months (Tables S16–S25), especially in two groups of consecutive months (one group included November, December, January, February and March, and the other group included August, September and October). However, comparing these two groups of consecutive months, we found that some pathways showed significant differences, including “endocytosis”, “dioxin degradation”, “D-Arginine and D-ornithine metabolism”, and “isoflavonoid biosynthesis”. The OTU abundances involved in these four different pathways were increased in the “August, September and October” group.

## Discussion

In the present study, it was demonstrated that bacterial composition and structure were highly diverse across the 12 months, indicating that environmental factors play crucial roles in shaping the composition of the milk microbiota.

The microbiota composition in March, June and July contained higher species richness, indicating that raw milk collected in these months was exposed to a greater risk of bacterial contamination (Fig. 1A). Coinciding with the temperature, it was shown that bacterial richness was generally low in winter and high in summer. However, the clusters of bacterial communities could be classified into two categories. It is easy to understand that the situations in spring and autumn are similar, but the bacterial structures in winter and summer were unexpectedly grouped together despite their large differences in bacterial richness (Fig. 4).

The phylum-level structure of the bacterial communities detected in the present study was similar to those found in previous studies^1^. However, an interesting phenomenon was observed that *Actinobacteria*, *Firmicutes* and *Proteobacteria* had counter-balanced relationships. Although the richnesses of these bacteria fluctuated over the year, the total proportion remained at a certain level (80%~95%), indicating that these microorganisms competed with each other for ecological niches.

Furthermore, the common genera previously detected in raw milk, such as *Pseudomonas*^22^, *Bacillus*^1^, *Lactococcus* and *Acinetobacter*^23^, were also identified in our samples. *Pseudomonas* was abundant in winter (November-March, average temperature 8.8 °C), which might be due to its psychrotrophic features. *Acinetobacter* was abundant in summer (June-September, average temperature 25.7 °C), since most species are widely distributed in nature and can survive at a broad range of temperatures. There was no obvious trend observed in *Lactococcus*, the richness of which changed randomly in adjacent months. It is probable that *Lactococcus* in raw milk might be affected by other environmental factors than temperature and humidity.

According to the alignment at the genus level, a tiny core microbiota (two genera, *Acinetobacter* and *Pseudomonas*) was observed. Only *Acinetobacter* was found to be consistent with the previously reported milk core microbiota (29 genera) after storage and transportation in tanker trucks^19^, hinting that the raw milk microbiota is easily subject to change by environmental factors rather than the consistent bacterial composition in tanker trucks or silos. Four phyla were abundant in all cow milk samples, including *Firmicutes*, *Proteobacteria*, *Actinobacteria* and *Bacteroidetes*. The four phyla showed more or less significant differences between months, which could be interpreted according to temperature and humidity changes. *Firmicutes* and *Proteobacteria* were very consistently correlated with high temperature and high humidity, respectively, while *Actinobacteria* and *Bacteroidetes* were the opposite (Fig. 7A).

Considering genus-genus correlations (Fig. 7C, Table S14, p < 0.01), *Lactobacillus* and *Bifidobacterium* had a strong positive correlation, which might be because both of them are main sources of probiotics and lactic acid producing strains. In addition, there might be a synergistic fermentation effect between these two genera. Meanwhile, both genera had positive correlations with high temperature and humidity. *Pseudomonas* (one of the four most abundant genera) had strong positive correlations with *Propionibacterium* and *Flavobacterium* (Fig. 7B), which could be due to their psychrotrophic characteristics. *Bacillus* (one of the four most abundant genera) had strong positive correlations with *Enhydrobacter*, *Enterococcus* and *Alkaliphilus*. Neither *Pseudomonas* nor *Bacillus* was correlated with humidity, but *Bacillus* had a positive correlation with temperature, while *Pseudomonas* had a negative correlation with temperature (Fig. 7B). Therefore, the moderate negative correlation (coefficient = −0.55, p = 0.06) between *Pseudomonas* and *Bacillus* might be mediated by temperature. Figure 7C also revealed positive correlations among the four genera *Niastella*, *Chitinophaga*, *Corynebacterium* and *Staphylococcus*, but no obvious consistency of these genera with temperature or humidity was observed (Fig. 7B). This result meant that some other environmental factors might affect the composition of bacterial populations, such as microbiota from teat apex^24^, milking equipment^25^, air, water^26^, etc.

With respect to the roles of the microorganisms in milk^1^, four kinds of microorganisms were confirmed, including genera that facilitate dairy fermentation (F), cause spoilage (S), promote health (H) and cause disease (D). In this study, genera with three of these roles (F, S and H) were abundant (Table S13). The genera *Salmonella*, *Campylobacter* and *Listeria*, which could cause disease, were identified in only five raw milk samples, and their proportion of the total microbiota was very low (<1E-4), coinciding with the strict quality control of cow breed and raw milk production toward opportunistic pathogens.

Since the genera *Pseudomonas* and *Bacillus* are known to play important roles in causing spoilage (S) after long periods of cold incubation^27^, we could easily conclude that raw milk from April, May, August, September and October would be subject to *Bacillus* spoilage, while the risk from *Pseudomonas* existed throughout the year (Table S13). The genera *Pseudomonas/Propionibacterium* and *Lactobacillus/Bifidobacterium* had strong positive correlations with each other, which could facilitate dairy fermentation (F)^1^. According to these results, we speculated that *Pseudomonas*/*Propionibacterium* and *Lactobacillus/Bifidobacterium* were two pairs of genera with synergistic effects in raw milk.

The interactive relationships of milk bacteria with raw milk parameters were elucidated. According to the correlation analysis, many genera significantly influenced raw milk quality, such as milk protein content, milk fat content, SCC, TBC, thermophilic spores, and psychrotrophic bacteria number.

A high SCC in raw milk usually indicated mastitis occurrence and problems in milking or general hygiene. It was reported that *Acinetobacter*, *Aeromonas* and *Brevundimonas* were persistent members of genera occurring in tap water^28^. This information provides an explanation for the implication of tap water bacteria in raw milk contamination. Meanwhile, *Aeromonas*, *Chryseobacterium* and *Stenotrophomonas* are opportunistic pathogens causing a broad spectrum of illnesses^29–31^. Therefore, the presence of these pathogens from udder, teats or wound infections might lead to an abnormal increase in SCC. The positive correlation between these genera and SCC (Table S15) suggested that there might be important pathogens within these genera.

Excessive bacterial count usually results from internal or external contamination^32^. The genus *Enterococcus* is an important contamination source and has been known in the dairy industry for decades^33^. As a risk or a foreign intrusive flora, the positive correlation between *Enterococcus* and TBC is suggestive of fecal contamination, indicating poor hygiene during milk handling and processing. However, *Propionibacterium* was found to be negatively correlated to TBC, which meant that it was helpful for maintaining the quality of raw milk. Dairy *Propionibacterium* was reported to be a candidate non-lactic acid probiotic, which predominantly ferments lactate to short-chain fatty acid (SCFA)^34^. Acetate and propionate have been shown to modulate immunity, reduce inflammation and protect against pathogens, and more importantly, to create an acid environment that inhibits the growth of other bacteria. This might be an explanation for its inhibitory effect.

*Pseudoalteromonas* was another genus negatively correlated to TBC. This genus of bacteria was reported to produce a broad range of anti-bacterial products, which have been found to aid them in colonization^35^. This might be the reason that it was able to limit TBC. It was suggested that opportunistic pathogens are inhibited by a cooperative network of bacterial communities, which are important in the maintenance of milk ecology. These findings led us to hypothesize that distinct interactive relationships existed in the milk microbiota.

Some genera were correlated with milk protein or milk fat, which are important nutritional parameters. *Bacteroides* and *Streptococcus* exist widely in the environment. In the raw milk production process, the lipolytic and proteolytic activities of these genera greatly contributed to the spoilage of raw milk.

Another important group of bacteria related to food safety and spoilage are the psychrotrophic bacteria, which could be detected in almost all natural environments. Due to their psychrotrophic nature and adaptability to different substrates and growth conditions, they can grow during refrigeration. It was estimated that psychrotrophic bacteria could cause a 10% loss of milk fats and proteins^36^. Because of their effects on milk quality, it is important that we develop sensitive and efficient tools to monitor the presence of these psychrotrophic bacteria. To our surprise, *Pseudomonas* was not found to be the major microorganism responsible for the degradation of milk properties, although it was one of the main genera identified in the raw milk microbiota. This might be another piece of evidence for the diversity of bacterial compositions in raw milk.

More genera were positively correlated to heat-resisting spore number, indicating that these genera themselves might be the main contributors of heat-resisting spores in raw milk, such as *Methylonatrum*, *Cloacibacillus*, and *Novispirillum*. In addition, the appearance of *Cloacibacterium* might indicate contamination with raw sewage.

Based on the microbial function predictions, some pathways differed between months, and some differences existed in adjacent months, such as March and April (Table S18), June and July (Table S21). On the other hand, some consecutive months showed no differences due to similar weather conditions, e.g., consecutive months November-March (average temperature 8.8 °C and humidity 71.8%) and consecutive months August-October (average temperature 23.9 °C and humidity 73.3%). A comparison between these two groups of consecutive months revealed that the OTU abundances involved in four pathways were highly increased in the high-temperature months (August-October), reflecting a more active metabolism in the microbiota during summer.

In most cases, management practices have a significant influence on milk quality. The importance of cow and stall hygiene and equipment hygiene for the quality of raw milk should be highlighted. More preventive actions related to environmental factors should be implemented to reduce the incidence of contamination.

## Materials and Methods

### Milk sources and sampling

In this study, 112 raw milk samples were aseptically collected from 10 different farms in Shanghai, China. All methods were carried out in accordance with the approved guidelines and regulations (GB 4789.1–2010, National Health and Family Planning Commission of the People’s Republic of China). Ten-milliliter milk samples were collected from the storage tanks of each farm and kept in sterile plastic tubes without preservative (Corning Life Sciences, USA). Then, the samples were immediately refrigerated at 4 °C, transported to the laboratory on ice, and frozen at −80 °C prior to DNA extraction. Samples were collected every month between July 2015 and June 2016.

### Genomic DNA extraction, PCR and 16 S rRNA gene sequencing

Bacterial genomic DNA was extracted by a Milk Bacterial DNA Isolation Kit (Norgen, Thorold, ON, Canada). The V3–4 region of the 16 S rRNA genes was amplified with primers 338 F and 806R^37^ with a random 8-bp bar code separately on the 5′ ends of 338 F and 806 R. PCR amplification was performed using TransStart FastPfu DNA Polymerase (TransGen, Beijing, China). The thermocycling steps were as follows: 5 min at 95 °C; 20 cycles of 45 sec at 95 °C, 30 sec at 55 °C, and 30 sec at 72 °C; and a final extension step for 10 min at 72 °C. Each sample was PCR amplified in triplicate. Then, the PCR products were purified with an AxyPrep DNA Gel Extraction kit (Axygen, Hangzhou, China) and pooled in equivalent amounts. The 16 S rRNA gene amplicons were sequenced on an Illumina MiSeq instrument^38^ at Shanghai Yuanxu Biotechnology Co., Ltd (China).

### 16 S rRNA gene sequence bioinformatics and statistical analysis

#### 16 S rRNA gene bioinformatic analysis

The raw data were separated by barcode using the MiSeq system program, and each sample was assigned paired FASTQ files. The paired FASTQ files were assembled using Mothur^39^ (version 1.37.0) with the command “make.contigs”. After removing sequences with ambiguous bases and shorter sequences (<350 bp), sequences were also excluded if they were identified as chimeric sequences or contaminants. The high-quality DNA sequences were grouped into OTUs with 97% by comparing them to the SILVA reference database (V119)^40^. Data normalization was carried out with the command “sub.sample” based on the minimum sample size (7,780). Community richness and diversity analysis (Shannon, ACE, Chao1 and Good’s coverage) were performed using the command “single.summary”. Taxonomy was assigned using the online software RDP classifier^41^ at an 80% threshold based on the Ribosomal Database Project^42^. The functional predictions based on the 16 S rRNA genes were performed using PICRUSt software.

#### 16 S rRNA gene statistical analysis

A t-test with 2 tails was used to determine whether the means of the Shannon diversity indices between June and Dec were significantly different using an R package, and p < 0.01 was considered a significant difference.

Abundance differences in genera between months were analyzed by Metastats^43^. Differences with q-values <1E-5 and fold changes >2 between months were considered significant, and the detailed results are listed in Tables S2–S12. The complex relations among the different genera were drawn using Circos software^44^ based on the major genera (percentage >1% in at least one month) and the phylum percentage. The coefficients of the relationships among genus abundances and between genera and milk quality parameters were analyzed using the non-parametric Spearman rank correlation with an R package. The p-value and adjusted p-value (q-value) were both calculated for finding relationships with a multiple test procedure^45^. Because most q-values were over 0.05 (some even 0.5), we chose p-value (p < 0.01) as a less stringent cutoff. The software Genesis (version 1.7.7)^46^ was used to draw heatmaps using the taxonomy information (genus information) of the representative OTU sequences, and hierarchical clustering was calculated for the monthly distributions. More differences between major genera are listed in Tables S2–S12. An RDA analysis based on the value of “axis lengths” (<3) was performed using the Vegan package in R. The identification of species was determined through BlastN based on the SILVA database and the NCBI NT database, and the parameters were set as identity ≥99% and alignment ≥97% based on a previous study^38^. To analyze the functional differences between months, a two-sided Welch’s test was performed using STAMP software, and the resultant P-values were multiply adjusted by the Benjamini-Hochberg procedure (q < 0.05); then, ratios of proportions higher than 2 were retained.

### Accession numbers

The sequence data have been submitted to the GenBank Sequence Read Archive (Accession number PRJNA389757).

## Electronic supplementary material

Table S1

Table S2-S12

Table S13

Table S14

Table S15

Table S16-S25
